# Evaluation of phytochemicals, antioxidant activity and amelioration of pulmonary fibrosis with *Phyllanthus emblica* leaves

**DOI:** 10.1186/s12906-016-1387-3

**Published:** 2016-10-24

**Authors:** Irsa Tahir, Muhammad Rashid Khan, Naseer Ali Shah, Maryam Aftab

**Affiliations:** 1 Department of Biochemistry, Faculty of Biological Sciences, Quaid-i-Azam University, Islamabad, 45320 Pakistan; 2 Department of Biosciences, COMSATS Institute of Information Technology, Islamabad, Pakistan

**Keywords:** *Phyllanthus emblica*, Pulmonary, Antioxidant, Histopathology, HPLC

## Abstract

**Background:**

In the present study the antioxidant potential of a methanol extract of *Phyllanthus emblica* leaves (PELE) was determined by in vitro methods as well as by an in vivo animal model, along with HPLC-DAD screening for phyto-constituents.

**Methods:**

The in vitro antioxidant potential of PELE was assessed by scavenging of DPPH, nitric oxide and anti-lipid peroxidation assays. For in vivo evaluation, a 60-day experimental plan was followed in which Sprague Dawley rats were administered with 1 mL/kg of CCl_4_ (CCl_4_ : DMSO + Olive oil; 30 % v/v) alone or with different doses of PELE (200, 400 mg/kg p.o.). Silymarin (100 mg/kg) as standard drug was also administered to CCl_4_ treated rats. HPLC-DAD analysis was performed to quantify polyphenolic phytochemicals.

**Results:**

PELE exhibited an appreciable in vitro antioxidant activity and scavenged the DPPH radical (IC_50_ = 39.73 ± 2.12 μg/mL) and nitric oxide (IC_50_ = 39.14 ± 2.31 μg/mL) while for anti-lipid peroxidation moderate antioxidant activity was noticed. Reduced levels of antioxidant enzyme activities viz., superoxide dismutase (SOD), catalase (CAT), glutathione peroxidase (GSH-Px) and reduced glutathione (GSH) whereas enhanced levels of total extractable proteins, lipid peroxides (TBARS), nitrite and H_2_O_2_ were induced by CCl_4_ administration in lungs of rat. Co-administration of PELE to rats exhibited a dose dependent decline in the oxidative injuries induced in these parameters. Histopathological damages such as disrupted alveoli, infiltration of macrophages and modified architecture of Clara cells was reversed to the normal state with co-administration of PELE. HPLC-DAD analysis indicated the presence of gallic acid, rutin, kaempferol and caffeic acid in the PELE.

**Conclusion:**

The findings of this study demonstrate that presence of polyphenolics and other active constituents in PELE might play a significant role in repairing the pulmonary damages instigated with CCl_4_.

## Background

Reactive oxygen species are produced as byproducts during the normal metabolic processes. Animal tissues are continuously handling these highly reactive byproducts which mainly include hydroxyl radical (^•^OH), superoxide anion (O_2_^−^) and hydrogen peroxide (H_2_O_2_) [1, 2]. Normally these reactive oxygen species (ROS) are produced in small amounts in the body for various physiological functions but if they are produced in excessive amounts, they can cause oxidative stress [3]. Free radicals are generally categorized into ROS and reactive nitrogen species (RNS) [4]. ROS/RNS have dual roles as they can be useful or harmful in biological processes [5]. ROS take part in various cellular signaling mechanisms. At physiological concentrations, ROS induce mitogenic responses but at higher concentrations to that of the physiological requirement these species may cause damage to the cellular organization by affecting membranes, proteins, lipids as well as nucleic acids [6]. Above all, cellular membranes are deleteriously affected by free radicals including reactive oxygen entities. The unsaturated fatty acids present in these membranes undergo peroxidation. The phenomenon of lipid peroxidation causes alteration in the chemical structure of the membranes possibly by the formation of cross links between adjacent lipids and by a decrease in the unsaturated/saturated ratio of fatty acids resulting in the decrease of membrane fluidity and consequently its function [7]. Hydroxyl radicals induce alterations in proteins responsible for generation of pathophysiological disorders such as atherosclerosis, cancer and neurological disorders. There are present certain antioxidant enzymes as well as non-enzymatic antioxidants which have the ability to cope with the deleterious effects of these free radicals [8].

Antioxidant activity is mechanistically complex involving either hydrogen atom transfer (HAT) and electron transfer (ET). Antioxidants displaying HAT show ability to quench free radicals by hydrogen donation. However, ET methods measure the ability of a potential antioxidant to abstract 1 electron from free radicals. Extracts/fractions are often composed of multiple active antioxidants with different chemical structures and polarity thus rendering diverse results depending upon the assay system in use. Taking into consideration different antioxidant assays there is no single assay by which total antioxidant capacity can be measured accurately and efficiently. Therefore, an approach of multiple assays in plant antioxidant potential is highly desirable. 1,1-Diphenyl-2-picryl-hydrazyl (DPPH) radical is frequently used for the antioxidant activity estimation of food as well as biological systems. DPPH is a stable radical which has an unpaired valence electron at one atom of nitrogen rendering it strong absorption at 517 nm. In the presence of hydrogen donor (free radical scavenging antioxidant) this unpaired electron becomes paired off and the resulting decolorization of DPPH correlates with the number of electrons captured [9]. Different studies have indicated that reactive nitrogen intermediates such as nitric oxide, peroxynitrite (ONOO^−^) and nitrogen dioxide (NO_2_) play crucial roles in the inflammatory process [10]. Younis et al. [11] reported that the extract/fractions of *Fraxinus xanthxyloides* inhibited the production of nitric oxide in LPS-activated RAW264.7 cells and in the carrageenan induced edema in rat.

Carbon tetrachloride (CCl_4_) is used in the living systems to mimics the oxidative stress-induced injuries. Metabolism of CCl_4_ via cytochrome P-450 resulted in different free radicals including trichloromethoxyl (CCl_3_O•) and peroxytrichloromethyl (CCl_3_OO•) radicals. These radicals can cause oxidative damages to pulmonary tissues by depleting the antioxidant enzymes and reduced glutathione (GSH) contents while elevating the lipid peroxides, nitrite content and hydrogen peroxide [12]. Oxidative stress induced by CCl_4_ in lungs of rat depleted the GSH reservoir through NADPH-dependent glutathione reductase and/or during glutathione dependent reduction of H_2_O_2_ and other peroxides via glutathione peroxidase [13]. Results of different studies indicated the beneficial role of plant extracts against pathophysiological alterations caused by free radicals after CCl_4_ metabolism [14]. The use of medicinal plants with an appreciable magnitude of antioxidant ability has been proposed as an effective therapeutic approach for pulmonary disorders.

On account of lower cost with no or minimum side effects, the population is becoming more inclined towards the use of complementary and alternative medicine. As a result natural compounds along with their derivatives are therapeutically evaluated for various ailments. Almost 50–60 % of all drugs used clinically world-wide are based on natural resources [15]. The fact that herbal remedies are now well acknowledged and well accepted, necessitates the evaluation of novel, effective and economical agents increasingly in order to fulfill the challenges concerning disease burden.

In recent decades, researchers have been trying to isolate and identify the natural antioxidants from plants [16] to scavenge the free radicals [17]. *Phyllanthus emblica* commonly called “amla” is one of them [18]. It belongs to the family Euphorbiaceae, and is a traditional Asian herbal drug used for the cure of different diseases including cancer, scurvy, and heart diseases [19]. *P. emblica* leaves are beneficial in cases of asthma, leucorrhoea, bronchitis, fever, and vomiting and are helpful in condition of biliousness and chronic dysentery [1]. Its fruit is used as a tonic to strengthen the body [20]. Khan et al. [21] reported that pyrogallol isolated from *P. emblica* as exhibiting promising activity against human lung cancer cells. The current experimental model was established to investigate the important pharmacological constituents in the leaves of the locally used therapeutic plant, *P. emblica*, collected from the region surrounding of Islamabad. Leaf extract was prepared in methanol and examined for polyphenolic constituents and antioxidant properties during in vitro assays and against CCl_4_ induced free radical production and pulmonary toxicity in rats.

## Methods

### Plant collection and extract preparation

The leaves of *P. emblica* were collected from the campus of Quaid-i-Azam University, Islamabad in 2013. The plant was identified at the Herbarium of Pakistan, Quaid-i-Azam University and assigned the voucher number of 61123. The collected leaves were rinsed with water, shade dried for 2 weeks at room temperature (20–25 °C) and then ground with the help of an electrical grinder. Two kg powder was macerated at room temperature with 4 liter of 95 % methanol for 36 h. The extraction was repeated twice and the filtrate was dried to obtain a solid residue by using rotary vacuum evaporator at 40 °C. The solid residue (PELE) was then stored at 4 °C to execute in vitro as well as in vivo evaluation. The total phenolic and total flavonoid content, different in vitro antioxidant assays and the various enzymatic and biochemical parameters of the lung samples were estimated by UV/VIS spectrophotometer.

### Total phenolic content (TPC)

For estimation of TPC in PELE, an aliquot of 100 μL of the test sample (1 mg/mL of methanol) was mixed with 1 mL of distilled water for dilution. Later, the reaction mixture was kept at 37 °C for 5 min after the addition of 1 mL (diluted 10 fold with distilled water) of Folin-Ciocalteu phenol reagent. After incubation period, 2 mL of distilled water with 1 mL of sodium carbonate (7 %) was added and reaction mixture was incubated once more for about one and half hour at 25 °C and absorbance was measured at 750 nm. Total phenolic content in PELE was estimated with reference to gallic acid (standard). TPC was measured in terms of mg GAE/g of extract.

### Total flavonoid content (TFC)

For the estimation of total flavonoid content in PELE, an aliquot of 100 μL (1 mg/mL of methanol) was added to the reaction mixture comprising of 0.5 M sodium nitrite (50 μL), 0.3 M aluminum chloride (50 μL) and 1 mL of 30 % methanol. Sodium hydroxide (300 μL of 1 M) was added in the reaction mixture. After keeping 10 min at room temperature, the absorbance was recorded at 546 nm.

### HPLC analysis

Chromatographic analysis was carried out by using Agilent RP-C8 analytical column attached to HPLC-DAD. Mobile phase A was acetonitrile-methanol-water-acetic acid (5:10:85:1) and mobile phase B was acetonitrile-methanol-acetic acid (40:60:1). A concentration gradient of 0 to 50 % of mobile phase B for 0–20 min, 50 to 100 % for 20–25 min and then 100 % B until 40 min was followed. The flow rate was 1 mL/min and injection volume was 20 μL. Standards and plant extract stock solutions were prepared in methanol, at a concentration of 200 μg/mL and 10 mg/mL respectively. Samples were filtered through 0.45 μm membrane filter. Nine reference standards i.e., catechin, rutin, kaempferol, quercetin, gallic acid, salicylic acid, apigenin, myricetin and caffeic acid (Sigma company, USA) were run at appropriate wavelengths. The PELE was analyzed at 257, 279, 325 and 368 nm wavelength. The column was reconditioned for 10 min before each run. All samples were assayed in triplicate. Quantification was carried out by the integration of the peak using the external standard method. All chromatographic operations were carried out at 20–25 °C temperature.

### In vitro antioxidant assays

#### DPPH radical scavenging activity

Radical scavenging action of PELE against the stable 1, 1-diphenyl-2-picrylhydrazyl radical was measured spectrophotometrically [22]. In order to measure the DPPH scavenging activity of PELE, DPPH solution was prepared by adding DPPH (12.5 mg) in 50 mL of methanol. Absorbance of this stock solution was measured at 517 nm. The DPPH solution was diluted with methanol to an absorbance of 0.98 and stored in an amber colored bottle ready for use. An aliquot of 100 μL of PELE at different concentrations (20, 40, 60, 80 and 100 μg/mL prepared in methanol) was added to 1 mL of DPPH solution. The reaction mixture was incubated at 37 °C in darkness for 20–30 min. Decrease in absorbance was determined at 517 nm. As a standard, ascorbic acid was used. The reaction was performed in triplicate. Scavenging activity was the calculated by using the Eq.1; where Q represents the DPPH scavenging activity.

1 $$ \mathrm{Q}\ \left(\%\right)=\frac{\mathrm{Absorbance}\ \mathrm{of}\ \mathrm{control} - \mathrm{Absorbance}\ \mathrm{of}\ \mathrm{sample}}{\mathrm{Absorbance}\ \mathrm{of}\ \mathrm{control}}\times 100 $$

### Nitric oxide scavenging assay

Nitric oxide scavenging activity of PELE was estimated by using Griess reagent as the main ingredient [23]. For this purpose an aliquot of 100 μL of the test sample (20, 40, 60, 80 and 100 μg/mL in methanol) was mixed with 100 μL of sodium nitroprusside (10 mM) prepared in saline phosphate buffer. The reaction mixture was kept at room temperature (20–25 °C) for 3 h and absorbance was recorded at 546 nm after adding 1 mL of Griess reagent. The nitric oxide scavenging activity of PELE was estimated by using equation 1; where Q represents the nitric oxide scavenging activity.

### Lipid peroxidation assay

Lipid peroxidation was determined with the help of the Ruberto et al. [24] protocol. For the quantification of lipid peroxide, lipid rich media was required which was prepared as egg yolk homogenate. A volume of 0.5 mL of the egg yolk (10 %) homogenate was mixed with 0.1 mL of PELE (20, 40, 60, 80, and 100 μg/mL) and volume increased by addition of 2 mL distilled water. Thereafter, 0.05 mL of FeSO_4_ (0.07 M) was added and the reaction mixture was heated at 37 °C for 30 min. Then, 1.5 mL of acetic acid (20 %) was added, and followed by the addition of 1.5 mL of TBA (0.8 %), prepared in 1.1 % sodium dodecyl sulphate. The reaction mixture was thoroughly mixed and place in tubes that were heated for 60 min in boiling water bath followed by the addition of 5.0 mL of butanol and centrifugation (3000 *g*) for 10 min. As a result, pink color was developed. Absorbance was measured at 532 nm and inhibition (%) was determined by the use of equation 1; where Q represents the anti-lipid peroxidation activity.

### In vivo experimental design and animal modeling

Forty two Sprague Dawley male rats (150–200 g) were kept at the Primate Facility present in Quaid-i-Azam University. The animals were placed in conventional steel cages at the room temperature (20–25 °C) with standard 12 h light and dark cycle. Normally, they were supplied with usual diet (rodent chow and tap water) but were fasted for 24 h before conducting the experiment. The experimental protocol was permitted by Ethical Committee of the Institute (Bch = 0249). The rats were treated by following the previously published protocol [25]. Seven groups, each containing 6 rats, were distributed as follows:

Group I (Sham control): Untreated; only standard food supply was offered. Group II (vehicle control): DMSO: Olive oil (1: 10 v/v) was administered intraperitoneally at 1 mL/kg body weight (bw). Group III: mixture of 30 % CCl_4_ in vehicle was given intraperitoneally (1 mL/kg bw), Group IV was treated with CCl_4_ solution in the same manner along with silymarin solution (100 mg/mL in vehicle) given orally. Group V and Group VI was administered with the CCl_4_ as above along with PELE (200 and 400 mg/kg bw p.o. respectively). Group VII received only PELE p.o. at 400 mg/kg bw. PELE was prepared at 100 mg/mL and 200 mg/mL in vehicle. The treatment of rats with CCl_4_ and the respective drug was administered on alternate days for 60 days.

After last treatment rats were unfed for 24 h. Chloroform was used to anesthetize the animals which were then dissected from ventral side. Blood was collected for both serum and whole blood analysis. The lungs were removed from the dissected animals and placed in saline solution. For histology, one of the lungs was stored in 10 % formalin solution while the other was stored in liquid nitrogen.

### Antioxidant enzymes evaluation

Homogenate (10 X) of lung samples was prepared by the addition of 10 volume of potassium phosphate buffer (pH 7.0). The homogenate was centrifuged for 30 min at 1500 *g* at temperature of 4 °C to achieve the supernatant and the following antioxidant enzymes assays were performed.

### Catalase (CAT) activity

For the determination of catalase activity, the method of Chance and Maehly [26] was followed. Tissue homogenate supernatant (25 μL) was mixed with 625 μL of 50 mM potassium phosphate buffer (pH 5.0) and 100 μL of 5.9 mM of H_2_O_2_ for one min and then its absorbance was measured at 240 nm. The catalase activity was described as an absorbance change of 0.01/min as one unit CAT activity.

### Superoxide dismutase (SOD) activity

In order to assess the activity of SOD, protocol of Kakkar et al. [27] was used. Assessment of SOD activity was determined by using 100 μL of 186 μM phenazine methosulphate and 300 μL of 17 mM sodium pyrophosphate buffer (pH 8.3). Tissue homogenate was first centrifuged at 1500 *g* for 10 min and then at 10,000 *g* for 15 min to get the enzyme in upper layer. Supernatant was gathered and 150 μL of it was added to the aliquot containing 600 μL of 0.052 mM of sodium pyrophosphate buffer (pH 7.0) and 50 μL of 186 μM of phenazine methosulphate. Finally, 100 μL of 780 μM of NADH was added to initiate enzymatic reaction. Addition of glacial acetic acid (500 μL) after 1 min stopped the reaction. Absorbance was determined at 560 nm to quantify the color intensity. Results were expressed in units/mg protein.

### Glutathione peroxidase (GSH-Px) Activity

GSH-Px activity was determined by implementing the Mohandas et al. [28] method. An aliquot of 50 μL of tissue homogenate was mixed with 740 μL of sodium phosphate buffer (0.1 M; pH 7.4), 50 μL of 1 mM of sodium azide, 25 μL of glutathione reductase (1 unit/mL), 5 μL of 0.25 mM of H_2_O_2_, 25 μL of 1 mM GSH, 50 μL of 1 mM of EDTA and 50 μL of 0.2 mM of NADPH. Oxidation of substrate i.e., NADPH was determined at 340 nm. Activity of GSH-Px was calculated as amount of NADPH oxidized per min per mg protein with the aid of molar coefficient 6.23 × 10^3^/M/cm.

### Estimation of reduced glutathione (GSH)

Estimation of reduced glutathione was done according to method of Jollow et al. [29]. Tissue homogenate (500 μL) was precipitated by the inclusion of 4 % sulfosalicylic acid (500 μL). After keeping for 1 h at 4 °C, the samples were centrifuged for 20 min at 1200 *g*. The supernatant (33 μL) was gathered and added to aliquots consisting of 900 μL of 0.1 M potassium phosphate buffer (pH 7.4) and 66 μL of 100 mM dithiobis (2-nitrobenzoic acid) (DTNB). GSH reacts with DTNB and produces a yellow colored complex. Absorbance was measured at 412 nm.

### Estimation of lipid peroxidation (TBARS assay)

The assessment of lipid peroxidation was carried out by using the method of Iqbal et al. [30]. The reaction mixture was consisted of 290 μL of 0.1 M phosphate buffer (pH 7.4), 10 μL of 100 mM of ferric chloride, 100 μL of 100 mM of ascorbic acid, and 100 μL of homogenized sample was incubated for 1 h at 37 °C in a shaking water bath. For quenching the reaction, addition of 0.5 mL of trichloroacetic acid (10 %) was made. The reaction tubes were then placed in the water bath for 20 min after adding 0.5 mL of thiobarbituric acid (0.67 %). Afterwards, the tubes were placed in crushed ice bath for 5 min and were centrifuged at 2500 *g* for 12–15 min. Absorbance was read at 535 nm against a blank. Results were accounted as nM of TBARS formed per min per mg tissue at 37 °C by using molar extinction coefficient of 1.56 × 10^5^ /M/cm.

### Hydrogen peroxide assay

Pick and Keisari [31] protocol was pursued to estimate the hydrogen peroxide via horseradish peroxidase mediated peroxidation of phenol red. In this activity assay, to 500 μL of 0.05 M phosphate buffer (pH 7) was added 100 μL of homogenate along with 100 μL of 0.28 nM phenol red solution, 250 μL of 5.5 nM dextrose, and horse radish peroxidase (8.0 units) was added and then incubated at room temperature for 60 min. In order to quench the reaction, 100 μL of 0.3 M NaOH was added and then mixture tubes were centrifuged for 5–10 min at 800 *g*. The absorbance of the collected supernatant was measured against reagent as a blank at 610 nm. The amount of H_2_O_2_ produced was expressed as nM H_2_O_2_/min/mg tissue on the basis of standard curve of H_2_O_2_ oxidized phenol red.

### Nitrite assay

For the accomplishment of nitrite assay, Griess reagent was utilized [32]. Equal quantities i.e., 100 μL each of 5 % ZnSO_4_ and 0.3 M NaOH were used to remove the proteins of tissue samples (100 mg), which were then centrifuged at 6400 *g* for 15–20 min. Later, 20 μL from the obtained supernatant was mixed with Griess reagent (1.0 mL) in cuvette and absorbance was recorded at 540 nm. Griess reagent (1.0 mL) was used to blank the spectrophotometer. The quantity of nitrite in the lung samples was estimated by measuring the absorbance of standard solution of sodium nitrite salt treated in the same way with Griess reagent.

### Histopathological investigation of tissues

The fresh samples of lung were sliced into small pieces and fixed in 10 % formalin for 3–4 h. The fixed tissues were rinsed in the course of an ascending sequence of alcohol (50, 70, 90 and 100 %). After clearing of tissues, the blocks were prepared by embedding the tissues in paraffin. For slide preparation a 3–4 μm thin section of the tissue was taken and stained in haematoxylin and eosin. In the end, slides were photographed by using light microscope with a camera attached (DIALUX 20 EB) at 40X.

### Statistical analysis

The experimental data of the in vitro assays was collected and analyzed by using the computerized GraphPad Prism software 5.0 so that the IC_50_ can be determined. In order to find out the effect of different treatments given to animals in the in vivo studies, one way analysis of variance was performed using Statistix 8.1 for Windows. The comparison among the treatments was made by using the Tukey’s multiple comparison test at probability level of ≤ 0.01.

## Results

### Phenolic and flavonoids content in PELE

Total phenolic content of PELE estimated was 195.8 ± 2.85 mg GAE/g dry extract while total flavonoids content was 346.2 ± 3.11 mg rutin/g dry extract.

### HPLC analysis of PELE

**Fig. 1:**
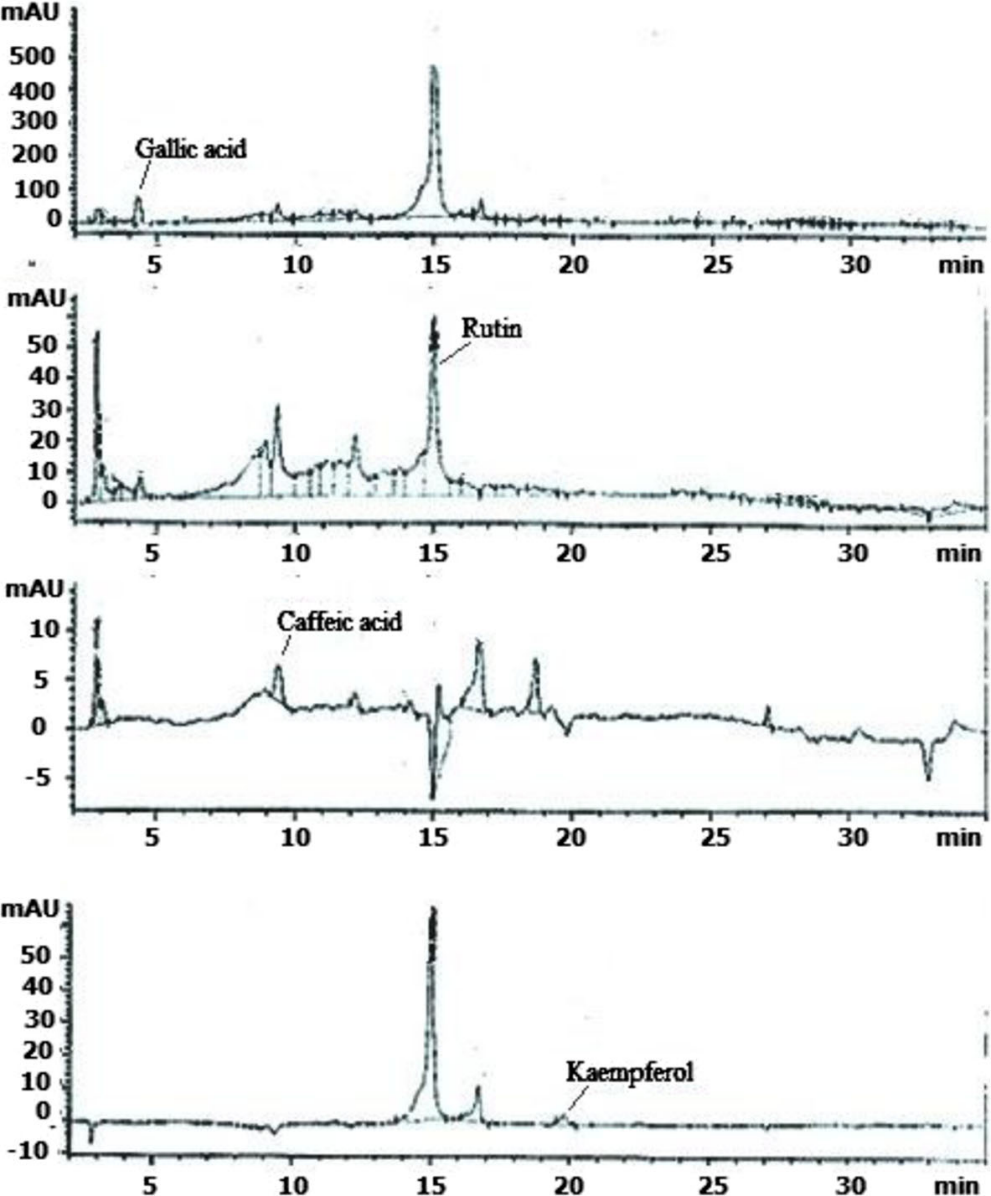
HPLC-DAD chromatogram of PELE

The polyphenolics constituents present in the PELE are presented in Table 1 and the chromatogram is shown in Fig. 1. Gallic acid constituted 1.10 μg/mg of the dry weight of extract. The quantities of other polyphenolics estimated were 113.8 μg/mg (rutin), 1.49 μg/mg (caffeic acid) and 1.54 μg/mg (kaempferol) of the dry weight of extract (Table 1).

**Table 1:** HPLC-DAD profile of standards and PELE

Reference flavonoids/phenolics	Signal wavelength	Retention time (min)	Regression analysis	R^2^	PELE (μg/mg dry extract)
Gallic acid	257	4.409	y = 3.15x + 7.84	0.92	1.10
Rutin	257	14.99	y = 1.78x-35.74	0.93	113.8
Catechin	279	7.237	y = 1.875x + 6.5	0.99	-
Caffeic acid	325	9.445	y = 12.4x + 23.4	0.96	1.49
Apigenin	325	21.757	y = 12.8x + 76.54	0.97	-
Myricetin	368	15.255	y = 2.7x-13.5	0.94	-
Quercetin	368	18.244	y = 4.9x + 31.13	0.99	-
Kaempferol	368	19.805	y = 11.78x + 120.24	0.93	1.54

**-**; not detected

### In vitro assessment of antioxidant potential of PELE

**Fig. 2:**
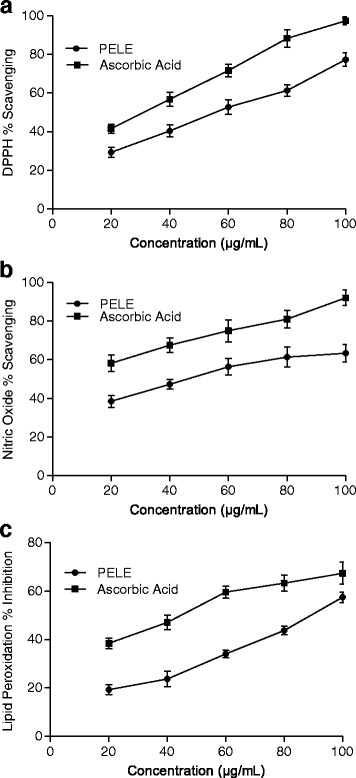
Dose response curve of PELE and ascorbic acid for in vitro antioxidant assays. **a** DPPH scavenging assay, **b**; Nitric oxide scavenging assay, **c** Lipid peroxidation scavenging assay. Each value corresponds to mean ± SD (*n* = 3)

**Fig. 3:**
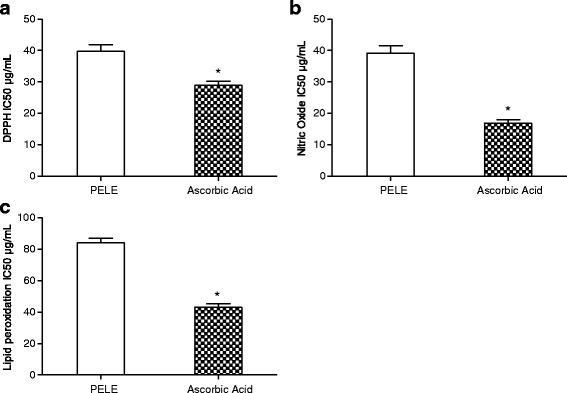
Medium inhibition concentration (IC_50_ μg/mL) of PELE for in vitro antioxidant assays. **a** DPPH scavenging assay, **b** Nitric oxide scavenging assay, **c** Lipid peroxidation inhibition assay. Each value corresponds to mean ± SD (*n* = 3). *; indicate significance at *P* < 0.01

Prior to the in vivo assessment of PELE, a variety of in vitro assays were performed. In order to determine and distinguish the antioxidant capability of plant extract (Fig. 2), IC_50_ estimation for the scavenging of different radicals can be considered as a valuable parameter. Figure 3 presents the IC_50_ values of PELE for different scavenging activity assays.

### DPPH free radical scavenging activity

In this experiment, it was employed for investigation of the antioxidant abilities of PELE. A dose dependent increase in percentage inhibition by plant extract was recorded when concentration increased from 20 to100 μg/mL (Fig. 2a). In the case of DPPH free radical scavenging assay, medium inhibitory concentration (IC_50_) value obtained for standard, ascorbic acid was 28.91 ± 1.35 μg/mL while plant extract had an IC_50_ value of 39.73 ± 2.12 μg/mL (Fig. 3a).

### Nitric oxide scavenging activity

The results obtained in this study indicated that percent inhibition of nitric oxide increased with an increase of the PELE and the ascorbic acid (20–100 μg/mL) concentration (Fig. 2b). The IC_50_ value of the ascorbic acid (16.89 ± 1.04 μg/mL) was significantly (*P* < 0.01) low as compared to the IC_50_ value (39.14 ± 2.31 μg/mL) of PELE (Fig. 3b).

### Lipid peroxidation assay

Figure 2c represents the dose response curve of PELE and the ascorbic acid on the percentage inhibition of lipid peroxidation. Use of PELE and ascorbic acid has shown dose dependent increase in percent inhibition of lipid peroxidation. The IC_50_ value of ascorbic acid (43.29 ± 2.07 μg/mL) was significantly (*P* < 0.01) low as compared to the IC_50_ value (84.10 ± 3.04 μg/mL) of PELE (Fig. 3c).

### Pulmonary protecting effect of PELE

In order to estimate the injuries caused by CCl_4_ to lung tissues and the extent of their recovery made by PELE, different assays were performed. The central objective of conducting this experiment was to estimate the protective ability of *P. emblica* leaves extract against the experimentally induced pulmonary toxicity.

**Fig. 4:**
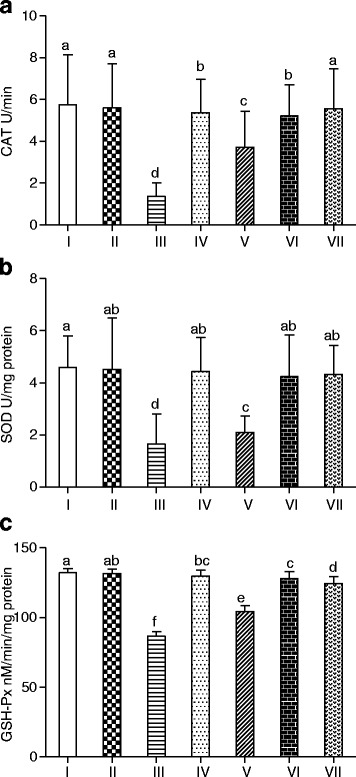
Graphical presentation of repairing potential of PELE against the toxicity of CCl_4_ in lungs of rat. **a** Catalase activity level, **b** Superoxide dismutase activity level, **c** Glutathione peroxidase activity level in pulmonary samples of rat. I; Control (untreated group), II; vehicle treated group, III; CCl_4_ treated group, IV; CCl_4_ + silymarin (100 mg/kg), V; CCl_4_ + PELE (200 mg/kg), VI; CCl_4_ + PELE (400 mg/kg), VII; PELE (400 mg/kg). Each value corresponds to mean ± SD (*n* = 3). Bars with different letters indicate significance at *P* < 0.01

The levels of CAT, SOD and GSH-Px activities are presented in Fig. 4. Results revealed that the level of these antioxidants was least in test group (administered by CCl_4_) and highest in the PELE co-administered groups. Comparable to control group, substantial reduction in SOD, CAT and GSH-Px activity level was detected in CCl_4_ treated group (III). This abnormality was erased in a dose dependent way by PELE treatment and values obtained were close to that of the control group. Moreover, a marginal difference (*P* > 0.05) was observed between the control group and only plant extract administered group in case of the pulmonary enzymes status.

The degree of lung damage was appraised by determining the protein, TBARS, nitrite and H_2_O_2_ contents (Table 2). As a result of CCl_4_ toxicity, protein and GSH contents of pulmonary samples of rat showed reduction while the TBARS, nitrite and H_2_O_2_ concentrations were observed to be elevated (*P* < 0.01). PELE administration to rats reversed the level of pulmonary protein, TBARS, nitrite as well as H_2_O_2_ towards the control group in a dose-dependent manner. The high dose of plant extract (400 mg/kg) gave healthier results as compared to low dosage (200 mg/kg). No significant distinction was observed between group I (control) and VII (plant extract only).

**Table 2:** Defensive effect of PELE on tissue proteins, GSH, TBARS, nitrite and H_2_O_2_ contents in lungs of rat

Treatment	Protein (μg/mg tissue)	GSH (nmole/min/mg protein)	TBARS (nmole/mg protein)	Nitrite (μM/mL)	H_2_O_2_ (nmole/mg tissue)
Control	2.5 ± 0.4^a^	22.0 ± 1.5^a^	3.1 ± 0.3^f^	34.5 ± 2.5^f^	1.03 ± 0.1^f^
Vehicle	2.4 ± 0.6^a^	21.6 ± 2.6^a^	3.9 ± 0.8^e^	39.4 ± 2.0^e^	1.25 ± 0.3^e^
CCl_4_ (1 mL/kg)	0.4 ± 0.5^d^	8.5 ± 2.2^d^	6.9 ± 1.1^a^	59.2 ± 2.5^a^	3.43 ± 0.3^a^
CCl_4_ + silymarin	2.2 ± 0.3^ab^	19.7 ± 2.4^b^	4.6 ± 0.4^b^	43.2 ± 1.2^c^	2.30 ± 0.4^b^
CCl_4_ + PELE (200 mg/kg) bw)	1.2 ± 0.16^c^	15.8 ± 1.2^c^	4.4 ± 0.9^bc^	45.0 ± 1.6^b^	2.17 ± 0.5^c^
CCl_4_ + PELE (400 mg/kg) bw)	2.1 ± 0.8^b^	19.7 ± 1.5^b^	4.1 ± 0.6^de^	41.6 ± 2.1^d^	1.95 ± 0.2^d^
PELE (400 mg/kg)	2.3 ± 0.7^ab^	20.9 ± 1.5^ab^	4.2 ± 0.5^cd^	42.1 ± 1.0^cd^	2.06 ± 0.3^cd^

Mean ± SD (n = 6), ^a-f^ (Means with different superscript letters) specify significance at *P* < 0.01, PELE; methanolic extract of *P. emblica* leaves

### Histopathology of pulmonary tissues

**Fig. 5:**
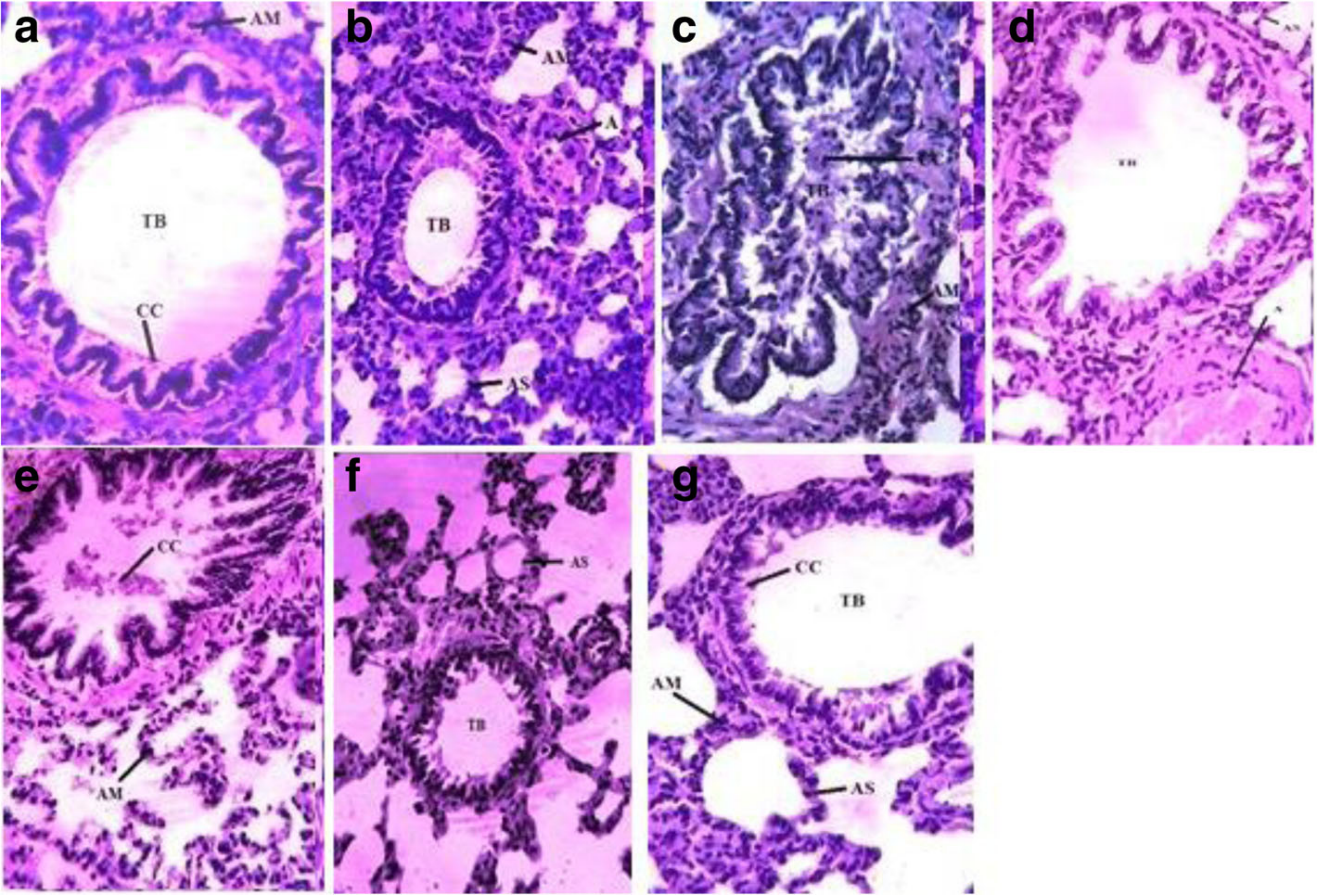
Microphotograph (40X) of H & E stained rats’ lung tissues. **a** Control (untreated group), **b** vehicle treated group, **c** CCl_4_ treated group, **d** CCl_4_ + silymarin (100 mg/kg), **e** CCl_4_ + PELE (200 mg/kg), **f** CCl_4_ + PELE (400 mg/kg), G; PELE (400 mg/kg). PELE; methanolic extract of *P. emblica* leaves, TB: terminal bronchiole, AS: alveolar septum, CC: Clara cells, AM: alveolar macrophages, A: arteriole

Histological inspection of pulmonary tissues was carried out after hematoxylin-eosin staining underneath the light microscope. Lung tissues from each experimental group were studied as shown in Fig. 5. Normal regular morphology of pulmonary tissues was observed in rats of control groups. Alveoli were thin walled with definite alveolar septa between adjacent cells. Regular fibroblasts were also noticed. The terminal bronchiole was of normal shape while the central cavity of bronchiole was rich with Clara cells. A clear cut difference in histology of CCl_4_ treated tissues was observed, when compared to normal rat assembly. CCl_4_ administration stimulated the degeneration process in alveolar septa along with distortion in elastic fibers and connective tissues. In addition, CCl_4_ caused clogging of blood capillaries and resulted in blood cells aggregation. Hindrance in breathing passage was the result of disorganization in Clara cells and inner epithelium of alveolar bronchioles. Administration of PELE decreased the toxicity and the resultant damages in pulmonary tissues, caused by CCl_4_. Ameliorating effects of PELE were more prominent at higher dosage. Normal alveoli with explicit alveolar spaces and bronchioles having slight cell degeneration were observed in most lung regions. However, even now less distinct intra-alveolar septa thickening was observed in few regions.

## Discussion

Phytochemical screening test of the crude methanol extract of *P. emblica* was executed and confirmed the existence of phenolics in it. The flavonoids are the major group of primary antioxidants. So, phenolics can scavenge or stabilize the free radicals either by reduction process or by forming complex with oxidizing compounds which are substantially stronger than those of vitamin C & E [33].

It is well known that elevated levels of ROS are the major cause of cellular oxidative damages and thus are implicated in numerous human diseases most importantly cancer, cardiovascular diseases, diabetes mellitus, arthritis, neurological collapse, and age acceleration process. Some plant genera such as *Emblica*, *Nepeta*, *Rosa*, and *Peganum* exhibited high antioxidant activities [34].

In current study, methanolic leaf extract of *P. emblica* leaves showed good scavenging ability (IC_50_ = 39.73 ± 2.12 μg/mL) against the DPPH radical, when compared to the ascorbic acid standard antioxidant (IC_50_ = 28.91 ± 1.35 μg/mL). PELE exhibited dose dependent percent scavenging of DPPH radicals. The substances with ability of performing this reaction are believed to be the antioxidants and hence radical scavengers [35]. HPLC-DAD studies of PELE exhibited the presence of polyphenolic compounds gallic acid, rutin, caffeic acid and kaempferol which might play a role in DPPH radical scavenging activity [36]. Similarly, the IC_50_ values for nitric oxide (39.14 ± 2.31 μg/mL) and lipid peroxides (84.10 ± 3.04 μg/mL) scavenging was also evaluated for PELE. In each case, PELE demonstrated substantial scavenging effects as compared to the IC_50_ values of the relative reference compounds such as ascorbic acid (Fig. 3).

This study was designed to explore the possible impact of *P. emblica* leaves extract on the pre-administration of toxin i.e., CCl_4_ which is believed to be the cause of disorders in tissues including lungs via free radical production [7, 25]. The CCl_4_ mediated cirrhotic response was observed to be superficially related to lung toxicity in humans. Antioxidant enzymes of pulmonary tissues were observed to be greatly reduced (SOD = 1.65 ± 1.15 U/mg protein, CAT = 1.37 ± 0.64 U/min, GSH-Px = 86.8 ± 3.1 nM/min/mg protein) in experimental animals when compared to normal ones (SOD = 4.60 ± 1.2 U/mg protein, CAT = 5.75 ± 2.4 U/min, GSH-Px = 132.0 ± 3.0 nM/min/mg protein), after intake of CCl_4_ (Fig. 4). But its administration in the company of PELE, significantly (*P* < 0.01) reverses the effect of CCl_4_ and thus prevents the decline in enzymatic actions, caused by toxin ingestion [25]. This suggests the availability of phenolics and polyphenolic constituents in *P. emblica* leaves. Likewise the pulmonary concentration of nitrite (59.2 ± 2.5 μM/ml) and H_2_O_2_ (3.43 ± 0.3 nM/min/mg) were increased (*P* < 0.01) with CCl_4_ administration as compared to the level of nitrite (34.5 ± 2.5 μM/ml) and H_2_O_2_ (1.03 ± 0.1 nM/min/mg tissue) in the untreated group. However, in the lungs of rats treated with CCl_4_ and PELE together the level of nitrite and H_2_O_2_ decreased in a dose dependent fashion. Polyphenolic constituents might be responsible for the observed protective effects in maintaining the levels of nitrite and H_2_O_2_ in CCl_4_ + PELE treated groups [25]. Similar results were reported by Khan et al. [14] while studying the defensive ability of extracts of *Sonchus asper* in rats against experimentally provoked pulmonary injuries.

In lungs of CCl_4_ treated rats the activity level of GSH (8.5 ± 2.2 nM/min/mg protein), a metabolite engaged in protecting organisms from oxidative injuries was low (*P* < 0.01) compared to control (22.0 ± 1.5 nM/min/mg protein) [37]. The lowered concentration of pulmonary GSH can be ascribed to the enhanced level of oxidative stress generated through CCl_4_ metabolism in the Clara cells of the lungs. The pulmonary injuries are attacked by the immune cells that in turn cause more oxidative injuries. The enhanced level of TBARS (6.9 ± 01.1 nM/min/mg protein) to that of control (3.1 ± 0.3 nM/min/mg protein) in lung samples treated with CCl_4_ reflected the peroxidation of polyunsaturated fatty acids [14]. Lipid peroxidation is a well-defined indicator of cellular damage caused by oxidative stress, in both animals and plants. During lipid peroxidation, a variety of toxic metabolites are generated. Lipid peroxides, involved in several pathological reactions, are unstable entities which disintegrate to form a series of compounds consisting of thiobarbituric acid reactive substances. PELE administration to CCl_4_ treated rats ameliorated the toxic effects on pulmonary tissues and the level of GSH and TBARS reverted towards the level of control animals. Further administration of PELE alone to rats did not induce significant alteration on the antioxidant enzymes and the GSH level to that of the control samples. These results suggested that PELE at this concentration (400 mg/kg) did not alter the basic antioxidant enzymes level implicating that PELE had not altered the function of immune cells residing in the pulmonary tissues [12].

ROS produced in normal physiological processes are considered as responsible agents for pulmonary injuries and fibrosis. The power of oxidative stress elicited by CCl_4_ against lung tissue is cleared by looking at the disturbed histological architecture which shows various injuries such as alveolar walls and bronchioles, aggregation of fibroblasts and disorganization of Clara cells was observed in the lung tissue [12]. Lungs are composed of different types of cells; epithelial cells, vascular endothelial cells, interstitial cells and cells of pulmonary defense system. Alveolar epithelium, neutrophils and macrophages are most active in the production of ROS. Edema and inflammatory responses of lungs are positively linked with the function of lungs, together with the oxygenation index and the airway pressure [38]. Lung injuries and inflammation can be attenuated by inhibiting the production of ROS. Similar toxic effects of CCl_4_ were also observed by Naz et al. [12] namely fibroblasts and inflammatory cell infiltration, significantly thicker inter-alveolar septa with collagen deposition, collapsed alveoli and severe epithelial degeneration. PELE treated rats showed marked reduction in epithelial degeneration and decrease in number of alveolar macrophages with suppression of inflammatory cellular infiltration. The protective effect of PELE might be due to the presence of polyphenolics in the extract, as anti-inflammatory properties have been reported for polyphenolics [39]. Ozyurt et al. [40] also reported similar results in which pulmonary fibrosis was inhibited by caffeic acid (3,4-dihydroxycinnamic acid) phenethyl ester, which is structurally related to flavonoids having free radical scavenging and antioxidant characteristics. HPLC-DAD analysis of PELE has indicated the presence of gallic acid, rutin, caffeic acid and kaempferol. Presence of gallic acid and rutin in leaves of *P. emblica* has been reported earlier [41]. Phytochemical investigations on *P. emblica* leaves have resulted in the isolation of the two new flavonoids, kaempferol-3-*O*-α-_L_-(6”-methyl)-rhamnopyranoside and kaempferol-3-*O*-α-_L_-(6”-ethyl)-rhamnopyranoside [42], acylated flavanone glycosides and phenolic glycosides [43]. The antioxidant potential of PELE in ameliorating the pulmonary toxic effects of CCl_4_ might be attributed by the presence of such constituents. Earlier studies reported the anti-inflammatory [44, 45] and antioxidant capabilities of rutin [46]. In a murine model of sepsis gallic acid was able to completely restore the lipid peroxidation whereas levels of CAT and SOD were partially restored [47]. The antioxidant potential of the extract obtained from the leaves of *P. emblica* is encouraging as improved medical treatments for oxidative stress induced lung disorders are strongly needed. It would be helpful in designing new medicines which are biologically more active and cost-effective with minimum side effects.

## Conclusion

In vitro antioxidant evaluation of the methanolic leaf extract of *P. emblica* leaves exhibited appreciable scavenging abilities for DPPH radical, nitric oxide and lipid peroxidation. The treatment of PELE to CCl_4_ exposed rat demonstrated strong repairing ability as manifested by the elevation in activity level of catalase, superoxide dismutase, glutathione peroxidase and GSH in the pulmonary samples of rat. PELE was also able to ameliorate the oxidative injuries induced with CCl_4_ and decreased the elevated level of TBARS, H_2_O_2_ and nitric oxide in lung samples of rat. The repairing abilities of PELE on the histopathology of lungs endorsed its antioxidant capabilities. The pharmaceutical potential of PELE might be attributed by gallic acid, rutin, caffeic acid, kaempferol and other active phyto-constituents. The results suggested that PELE has the ability to modulate cellular responses and maintain the functional integrity of pulmonary tissues.

## Abbreviations

CAT: Catalase
CCl_4_: Carbon tetrachloride
GSH: Reduced glutathione
H_2_O_2_: Hydrogen peroxide
PELE: *Phyllanthus emblica* methanol extract of leaves
SOD: Superoxide dismutase
TBARS: Thiobarbituric acid reactive content
